# Biodegradable Thermosensitive Hydrogel for SAHA and DDP Delivery: Therapeutic Effects on Oral Squamous Cell Carcinoma Xenografts

**DOI:** 10.1371/journal.pone.0033860

**Published:** 2012-04-18

**Authors:** Jing Li, Changyang Gong, Xiaodong Feng, Xikun Zhou, Xiaoping Xu, Liang Xie, Ruinan Wang, Dunfang Zhang, Hui Wang, Peng Deng, Min Zhou, Ning Ji, Yu Zhou, Yun Wang, Zhiyong Wang, Ga Liao, Ning Geng, Liangyin Chu, Zhiyong Qian, Zhi Wang, Qianming Chen

**Affiliations:** 1 State Key Laboratory of Oral Diseases, West China College of Stomatology, Sichuan University, Chengdu, China; 2 State Key Laboratory of Biotherapy and Cancer Center, West China Hospital, West China Medical School, Sichuan University, Chengdu, China; Indiana University School of Medicine, United States of America

## Abstract

**Background:**

OSCC is one of the most common malignancies and numerous clinical agents currently applied in combinative chemotherapy. Here we reported a novel therapeutic strategy, SAHA and DDP-loaded PECE (SAHA-DDP/PECE), can improve the therapeutic effects of intratumorally chemotherapy on OSCC cell xenografts.

**Objective/Purpose:**

The objective of this study was to evaluate the therapeutic efficacy of the SAHA-DDP/PECE in situ controlled drug delivery system on OSCC cell xenografts.

**Methods:**

A biodegradable and thermosensitive hydrogel was successfully developed to load SAHA and DDP. Tumor-beared mice were intratumorally administered with SAHA-DDP/PECE at 50 mg/kg (SAHA) +2 mg/kg (DDP) in 100 ul PECE hydrogel every two weeks, SAHA-DDP at 50 mg/kg(SAHA) +2 mg/kg(DDP) in NS, 2 mg/kg DDP solution, 50 mg/kg SAHA solution, equal volume of PECE hydrogel, or equal volume of NS on the same schedule, respectively. The antineoplastic actions of SAHA and DDP alone and in combination were evaluated using the determination of tumor volume, immunohistochemistry, western blot, and TUNEL analysis.

**Results:**

The hydrogel system was a free-flowing sol at 10°C, become gel at body temperature, and could sustain more than 14 days in situ. SAHA-DDP/PECE was subsequently injected into tumor OSCC tumor-beared mice. The results demonstrated that such a strategy as this allows the carrier system to show a sustained release of SAHA and DDP in vivo, and could improved therapeutic effects compared with a simple additive therapeutic effect of SAHA and DDP on mouse model.

**Conclusions:**

Our research indicated that the novel SAHA-DDP/PECE system based on biodegradable PECE copolymer enhanced the therapeutic effects and could diminished the side effects of SAHA/DDP. The present work might be of great importance to the further exploration of the potential application of SAHA/DDP-hydrogel controlled drug release system in the treatment of OSCC.

## Introduction

Oral squamous cell carcinoma (OSCC) is one of the most common malignancies leading to death that accounts for more than 90% of all oral cancers [1]. Although the aetiological risk factors are well documented and advances in diagnosis and therapy have been made in the different treatment modalities, the morbidity of OSCC have not improved significantly over the last decades [2], [3].

Histone deacetylase (HDAC) inhibitors have been shown to acetylate the nucleosomal histones of condensed chromatin, and cause the reactivation of genes silenced by hyperacetylated histones [4]. HDAC inhibitors, including suberoylanilide hydroxamic acid (SAHA), have demonstrated therapeutic benefit as monotherapy on a variety of hematological and solid tumor cancer such as glioma, head and neck cancer, hematologic malignancies and nonsmall-cell lung cancer (NSCLC) [5], [6], [7], [8]. Cisplatin (DDP) is another strong and widely used chemotherapy drug which is used to treat cancers including: sarcoma, small cell lung cancer, germ cell tumors, lymphoma, and ovarian cancer [9]–[14]. Though SAHA and DDP have been proven to be effective therapeutic efficacy, they are also can cause serious side effects. The conventional methods for delivering chemotherapeutic agents fail to achieve therapeutic concentrations of drugs, despite reaching toxic systemic levels. Moreover, the improvement of the curative is accompanied by the resistance to the drugs [15]–[18]. So, the novel controlled drug delivery systems are necessary designed to deliver drugs at predetermined rates for predefined periods at the target organ and overcome the shortcomings of conventional drug formulations, therefore could diminish the side effects and improve the life quality of the patients. Thus, a suitable controlled drug delivery system is extremely important for chemotherapy.

In our previous study, we prepared a new kind of biodegradable, temperature sensitive and injectable poly (ethylene glycol)-poly(ε-caprolactone)-poly(ethylene glycol) (PEG-PCL-PEG, PECE) hydrogel based on PEG and PCL which are biocompatible and have been used in several FDA approved products [19]. The PECE hydrogel was a flowing sol at low temperature and formed a non-flowing gel at body temperature, the sol-gel-sol transition behavior of it has been reported in our previously study. And the results indicated that PECE hydrogen displayed a temperature-dependent sol-gel-sol transition in normal saline. When the concentration was above corresponding critical gelatin concentration, the aqueous solutions of PECE copolymer undergo a sol-gel-sol transition as the temperature increases [20], [21]. PECE also was proved to be biocompatible, bioabsorbable and thermosensitive. The thermosensitive hydrogel system had been successfully used for honokiol, 5-Fu, and bFGF controlled delivery in cancer treatment and the results presented its great sustained drug release properties [22]–[25]. Based on the above results, the PECE hydrogel might have great potential applications as an injectable controlled drug delivery system.

In this report, we investigated the combined effects of HDAC inhibitors SAHA and DDP in an oral squamous cell carcinoma tumor model using the PECE hydrogels for controlled drug delivery. Our results suggested that the SAHA combine DDP delivered by PECE hydrogel have a significant therapeutic efficacy in murine model. This strategy had potential application in chemotherapy for the oral squamous cell carcinoma.

## Materials and Methods

### Animals and Cells

Female athymic nude mice (4–6 weeks) were obtained from the Shanghai SLAC laboratory animal co. ltd (Shanghai, China). All animals were handled in strict accordance with good animal practice as defined by the relevant national and/or local animal welfare bodies, and in accordance with the recommendations in the Guide for the Care and Use of Laboratory Animals of the National Institutes of Health. Animal experiments were approved by the Institutional Animal Care and Treatment Committee of Sichuan University (Chengdu, China).

Oral squamous cell carcinoma cells HSC-3 cells were maintained in Dulbecco’s modied Eagle’s medium (DMEM; Gibco, USA) containing 10% fetal calf serum (Gibco, USA), penicillin (100 U/L) and streptomycin (10 mg/L). HOK16E6E7 cells, a human immortalized oral keratinocyte cell line, was cultured in keratinocyte growth medium (KGM) containing 0.15 mM of calcium, supplemented with EGF (Gibco, USA), both at 37°C in an atmosphere containing 5% CO_2_.

### Reagents

SAHA was purchased from (Alexis Corp., San Diego, CA, USA), and was dissolved in DMSO as stock solution. The maximum volume (%) of DMSO in the experiment was less than 0.1%. DDP was purchased from Jintai Pharmaceutical Co. Ltd. (Liaoling, PR China) and dissolved in PBS. Poly (ethylene glycol) methyl ether (MPEG, Mn  =  550), ε-caprolactone (ε-CL), hexamethylene diisocyanate (HMDI), and stannous octoate (Sn(Oct)_2_) were purchased from Sigma-Aldrich Chemical Co. (USA).

### Synthesis of PECE Hydrogel

PECE copolymer were synthesized and purified as reported previously [19]. Briefly, PEG-PCL diblock copolymers were prepared by ring opening polymerization of ε-CL initiated by MPEG using stannous octoate as catalyst; PEG-PCL-PEG triblock copolymers were synthesized by coupling PEG-PCL diblock copolymers using HMDI as coupling agent. The obtained PECE copolymer was dissolved in AR grade dichloromethane and reprecipitated from filtrate using AR grade petroleum ether. Then the mixture was filtered and vacuum dried to constant weight at room temperature. The obtained PECE copolymers were characterized by FTIR (NICOLET 200SXV, Nicolet, USA), ^1^H-NMR (Varian 400 spectrometer, Varian, USA), and GPC (Agilent 110 HPLC, USA).

### Preparation of Injectable Thermosensitive Composite Hydrogel

The drugs loaded injectable thermosensitive hydrogel was prepared similar to the protocol reported previously [26], [27]. First, PECE copolymer was well dissolved in water at certain temperature and cooled to 4C to form sol. Then, SAHA and DDP solutions were mixed into the PECE sol to form homogeneous solution, and the concentration of PECE was kept at 30 wt%. The prepared drugs loaded hydrogel was inhaled into injector and injected into or around the focus of infection in animal. Thus, composite sol turned into gel state and acted as depots for sustained release of drugs *in situ* when the cold sol is warmed to body temperature (37°C) *in vivo*. At last, for the degradation of the composite hydrogel, the introduced DDS was gradually emanated from body.

### Cytotoxicity Assay of PECE Copolymer

Cytotoxicity of PECE triblock copolymer to HOK16E6E7 and HSC-3cells was evaluated by 3-[4, 5-dimethylthiazol-2-yl]-2,5-diphenyl tetrazolium bromide (MTT) assay. Cells were seeded on 96-well plates at a density of 10^4^ cells/well in their usual culture media. After 24 h, different amounts of PECE copolymer were added 0 µg, 10 µg, 20 µg, 50 µg, 100 µg, 150 µg, 200 µg, 300 µg, or 400 µg each well, respectively. The normal saline was used as the control groups. Cytotoxicity studies were performed using MTT assay after 24 h, 48 h or 72 h. The absorbance of the samples was measured at 570 nm. The cell cytotoxicity of PECE copolymer is defined as the relative viability, which is the ratio of the number of live cells to that of the control cells (100%).

### Murine Tumor Models and Treatment

The female nude mice were inoculated subcutaneously into the right flank regions with 2×10^6^ HSC-3 cells/mouse resuspended in 0.1 ml of DMEM medium without serum and antibiotics. When tumors were palpable one week later, the mice were randomly assigned to six independent treatment groups (6 mice per group): (a) mice treated with 100 µl normal saline (NS), (b) mice treated with 100 µl blank hydrogel, (c) mice treated with 100 µl SAHA, (d) mice treated with 100 µl DDP, (e) mice treated with 100 µl combine SAHA with DDP (SAHA-DDP), (f) mice treated with 100 µl SAHA and DDP in thermosensitive hydrogel (SAHA-DDP/PECE). SAHA and DDP were peritumorally administered two times at the first dose and 14 days later. Tumor diameters were measured every 3 days, and tumor volume was calculated using the formula: Tumor size  =  (length) × (width)^2^ × 0.52. All the animals were observed after administration, including the general conditions (the activity, energy, hair, feces, behavior pattern, and other clinical signs), body weight, and mortality. Mice were sacrificed 14 days after the last treatment. Tumors were excised and weighted, half of which were snap frozen immediately for western blot assay, and anthers were fixed in 4% formaldehyde.

### Toxicity Assessment

Possible side effects were observed through weight, appetite, diarrhea, life span, and behavior until they were sacrificed. Organs such as heart, liver, spleen, lung, and kidney were collected, fixed in 4% paraformaldehyde solution and made into 4 µm sections which were stained with hematoxylin and eosin (H&E) and observed by two pathologists in a blinded manner under a microscope.

### Immunohistochemistry and TUNEL Assay

Mouse anti-human PCNA antibody (1∶500, BD, USA) was applied for detecting the tumor cell proliferation on the paraffin section. Microvessel of tumor tissues was detected by Anti-CD34 antibody (1∶500, BD, USA) on lung frozen tissue section. TUNEL detection Analysis of apoptotic cells in tumor tissue was measured by the TUNEL Apoptosis Detection Kit (Upstate LabChem Inc, USA) following the manufacturer’s directions. Three equal-sized fields (at × 400 magnification) were randomly chosen and the mean number of green fluorescence-positive cells was counted.

### Western Blot Analysis

To prepare lysates from dissected in vivo tumors, samples were snap frozen in liquid nitrogen immediately after animal sacrifice and stored at –80°C before use. The lysates were incubated on ice in RIPA lyses buffer for 30 min before being homogenized by a mortar and pestle. Apoptosis related proteins were detected by anti-Caspase-3 antibody, anti-Caspase-8 antibody, anti-Caspase-9 antibody (Cell Signaling, USA). Isolation of histones was performed as described previously [28]. Total and acetyl-Histone H3 were detected by Anti-Histone H3 polyclonal antibody and Anti-acetyl-Histone H3 polyclonal antibody respectively. The primary antibody was detected with HRP-conjugated anti-mouse secondary antibody and developed with an enhanced chemiluminescence detection kit (Pierce, USA). Equal protein loading was confirmed by detection of GAPDH (R&D, USA).

### Statistical Analysis

Data are expressed as the mean ± SD. Statistical analysis was performed by Student’s t test for comparing two groups and by ANOVA for multiple group comparisons. *p* values less than 0.05 were considered to be statistically significant. All statistical tests were two-sided. SPSS 16.0 was used for all statistical analyses.

## Results

### Synthesis and Characterization of PECE Copolymers

**Figure 1:**
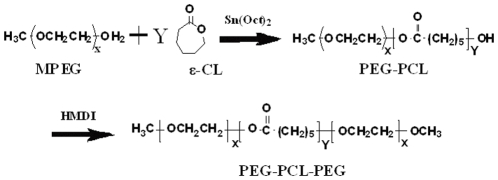
Synthesis of PECE copolymers.

The PECE triblock copolymer was synthesized according to Fig.1. FTIR, ^1^H-NMR and GPC were used to characterize the chemical structure of the PECE copolymer (data not shown) [19]. The Mn and PEG/PCL block ratio of PECE triblock copolymer calculated from ^1^H-NMR spectra was 3630 and 1100/2530 respectively.

### Cytotoxicity Assay of PECE Copolymer

**Figure 2:**
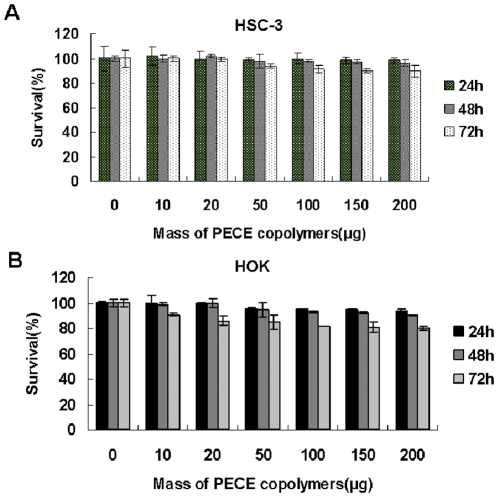
**Cytotoxicity Assay of PECE Copolymer.** Different amounts of PECE copolymer were added from 5 to 400 µg each well, and after 24, 48 and 72 hours, survival rate of HOK16E6E7 and HSC-3 cells was determined by MTT assay. Data were obtained from three independent triplicate experiments and were presented as mean ± S.D (*P<0.01).

Firstly, We wanted to verify whether the prepared PECE copolymer has cytotoxicity to the human oral normal and cancer cell line, so HOK16E6E7 (an immortalized human oral keratinocyte cell line) and HSC-3 ( a human tongue squamous cell carcinoma cell line) were chose in the cytotoxicity assay of PECE copolymers. Fig. 2 exhibits the HOK16E6E7 and HSC-3 cells viability at the presence of PECE copolymer at different concentrations. The cell viability decreased with increase of PECE copolymer amount. But the HOK16E6E7 and HSC-3 cells viability was yet higher than 82% and 65%, respectively, even when the input PECE copolymers were 400 µg per well. According to Fig. 2, the HSC-3 cell viability of PECE copolymer was higher compared to HOK16E6E7 group. Thus, the PECE copolymers prepared in this paper could be regarded as a biocompatible with low cell cytotoxicity controlled drug delivery systems.

### Antitumor Activity of SAHA-DDP/PECE In Vivo

In the mice model of oral squamous cell carcinoma xenografts, the female nude mice received 2 × 10^6^ HSC-3 cells via subcutaneously into the right flank regions. Mice were assigned to six groups, receiving NS, PECE, SAHA, DDP, SAHA-DDP and SAHA-DDP/PECE, respectively.

**Figure 3:**
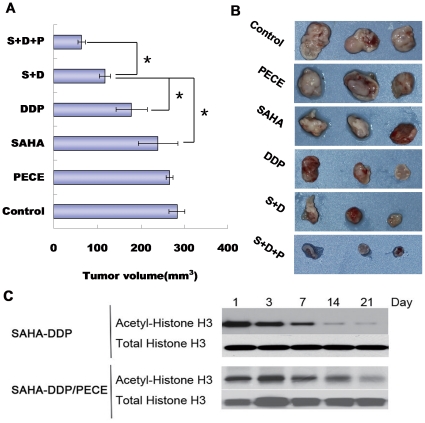
**Antitumor activity of SAHA-DDP/PECE in vivo.** In the mice model of oral squamous cell carcinoma xenografts, the female nude mice received 2 × 10^6^ HSC-3 cells via subcutaneously into the right flank regions. (A) The mice were then treated with NS, PECE, SAHA, DDP, SAHA-DDP and SAHA-DDP/PECE every two weeks for a total of two doses starting on day 7 (n = 6 mice per group). Tumor volumes of mice from different groups of HSC-3 tumor model. (B) Representative tumors of OSCC mice model from NS, PECE, SAHA, DDP and SAHA-DDP control group and SAHA-DDP/PECE treated mice. Data are representative of at least two separate experiments. Bars, means ± SD (*P<0.05).(C) When tumors were palpable, the mice were randomly assigned to two independent treatment groups( n = 12 mice per group): mice treated with 100 ml combine SAHA with DDP (SAHA-DDP), or treated with 100 ml SAHA and DDP in thermosensitive hydrogel (SAHA-DDP/PECE). At the date of 1, 3, 7, 14 and 21 three mice were sacrificed separately. The ribonucleoprotein of the tumor tissues was extracted and the expression of acetyl-Histone H3 and Histone H3 was detected by western blot.

Two combination-therapy groups, especially the group treated with SAHA-DDP/PECE were significantly reduced the growth of tumors compared with other control groups. The growth delay of tumors in mice injected with SAHA-DDP/PECE continued until the day of sacrifice, reaching a mean volume of 62.43 mm^3^, while tumors of mice injected with NS, PECE, SAHA, DDP and SAHA-DDP grew persistently, resulting in mean volumes of 148.60 mm^3^, 187.60 mm^3^, 249.71 mm^3^, 279.41 mm^3^ and 284.32 mm^3^ (Fig. 3B and C, p < 0.05). Consistent with the data of tumor volume, the mean weight of tumors of mice treated with SAHA-DDP/PECE was 0.04 ± 0.01 g, accounting for 22.18% of that of mice treated with NS (0.21 ± 0.10 g), 22.42% of mice treated with PECE (0.20 ± 0.04 g), 25.22% of mice treated with SAHA (0.18 ± 0.04 g), 33.54% of mice treated with DDP (0.14 ± 0.02 g) and 43.98% of mice treated with SAHA-DDP (0.10 ± 0.01 g).

To provide evidence that the drugs were detectable at the tumor site and the duration of drug presence following drug delivery, we have detected the expression of acetyl-Histone H3 in order to reflect the controlled releases ability of SAHA-DDP/PECE indirectly. As shown Fig. 3C, the level of acetyl-Histone H3 was observably reduced with the lapse of time in SAHA-DDP group while high expression level of acetyl-Histone H3 was maintained in the SAHA-DDP/PECE group in an extended period.

### Systematic Toxicity in the SAHA-DDP/PECE Treated Mice

**Figure 4:**
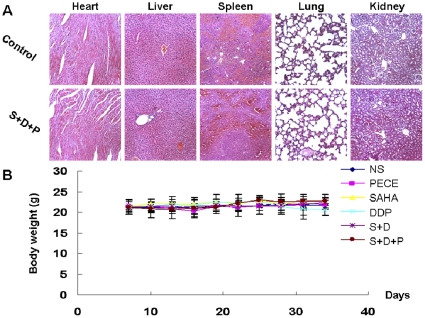
**Systematic toxicity in the SAHA-DDP/PECE treated mice.** H&E staining of section of major tissues obtained from tumor beard mice which received two doses of NS, PECE, SAHA, DDP, SAHA-DDP and SAHA-DDP/PECE. Heart, liver, spleen, lung and kidney were harvested at day 28 after intravenous injection. (A) Mean body weights on days 7, 10, 13, 16, 19, 22, 25 and 28 of mice treated with two doses of NS, PECE, SAHA, DDP, SAHA-DDP and SAHA-DDP/PECE; error bars correspond to 95% confidence intervals, Values are means±SD (n  =  10 mice per group).

Since earlier researches confirmed the severe irreversible side effects of DDP and SAHA treatment, we compared the body weight and other side effects on mice treated with free SAHA, DDP or SAHA-DDP/PECE. No difference of body weight was observed among the SAHA-DDP/PECE group compared with the other groups at the end of the study (Fig. 4A). After mice were sacrificed, their liver, lung, kidney, spleen and heart were harvested and H&E histological staining was performed. As observed by two pathologists in a blinded manner, we found that mice treated with SAHA-DDP/PECE showed no slight changes of toxicity to the organ tissues, compared with the normal organ tissues from mice receiving NS (Fig. 4B). In addition, no conspicuous adverse effects in gross measures were observed, such as appetite, feeding, ruffling of fur, behavior change, etc.

### SAHA-DDP/PECE Inhibited Cell Proliferation and Intratumoral Angiogenesis In Vivo

**Figure 5:**
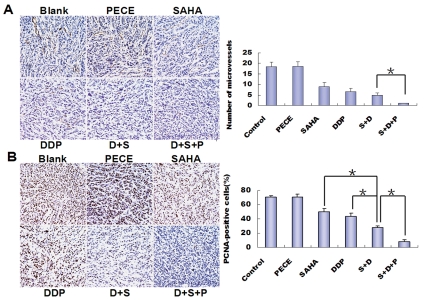
**SAHA-DDP/PECE inhibited cell proliferation and intratumoral angiogenesis in vivo.** (A) PCNA-positive cells were rich in NS and PECE groups. Whereas, the percentage of PCNA-positive cells in SAHA, DDP, SAHA-DDP and SAHA-DDP/PECE groups were significantly decreased in turn (*P<0.05). (magnification, × 200) (B) Angiogenesis within tumors was detected by CD34 staining of micro vessels. The average number of micro vessels per vascular hot spot was significantly decreased in SAHA-DDP/PECE treated tissues compared with those in the three control groups (*P<0.05).

To address possible mechanisms responsible for the in vivo antitumor activity of combination-therapy groups, we made an inquiry into phenotypic changes of tumor tissues in cell proliferation and intratumoral angiogenesis. We first performed PCNA staining to assess the effect of combination-therapy groups, suppression on tumor cell proliferation. Tumor tissues of two combination-therapy groups, especially the group treated with SAHA-DDP/PECE were exhibit much less staining for PCNA compared with that in tumor tissues of mice treated with NS, PECE, SAHA, DDP (Fig. 5A, p < 0.05). Some studies have shown that MVD and perimeter are significantly different between normal mucosa and OSCC [29]–[31]. Therefore, MVD was evaluated in the tumors harvested from mice. We examined the tumor tissues with anti-CD34 and the most highly vascularized area of each tumor was identified and five high-powered fields were counted in this area for MVD. As shown in Fig. 5B, the most significant reduction of MVD was observed in the group treated with SAHA-DDP/PECE (19.7±4.8) compared with the other groups. Thus, it may be hypothesized that the anti-tumor effects of SAHA-DDP/PECE could be contributed to its ability of inhibits cell proliferation and intratumoral angiogenesis. However, no significant difference was found among SAHA, DDP group and SAHA-DDP group.

### SAHA-DDP/PECE Induced Apoptosis In Vivo

**Figure 6:**
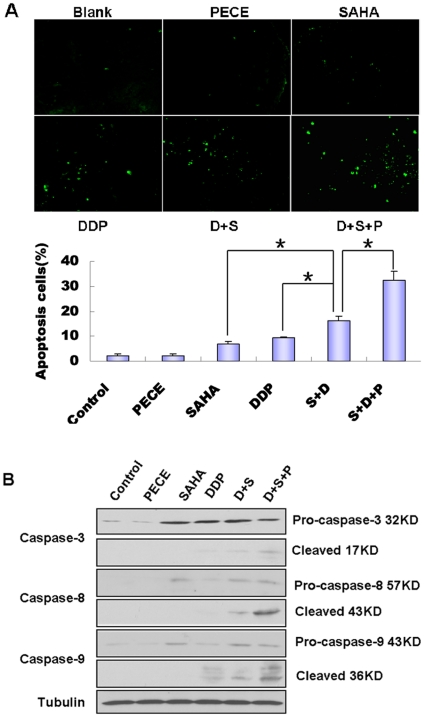
**SAHA-DDP/PECE induced apoptosis in vivo.** (A) Induction of apoptosis was indicated by TUNEL assay. The TUNEL-positive cells display dark green nuclei and are observed under a fluorescence microgroup (×400 magnification). TUNEL-positive nuclei were significantly increased in SAHA-DDP/PECE treated tissues compared with those in the control groups (*P<0.05). (B) Detection of caspase-3, Caspase-8 and Caspase-9 by Western blots. GAPDH was used as equal loading control.

The cytotoxic effects mediated by combination-therapy groups were also analyzed to evaluate whether the cytotoxic mechanisms of the constructs observed included the element of apoptosis in the HSC-3 tumor model. We performed the in situ TUNEL assay to evaluate the influence of combination-therapy groups on apoptosis in tumor cells. We observed an apparently elevated apoptosis rate in tumors of mice with combination-therapy, especially the group treated with SAHA-DDP/PECE compared with that in tumors of mice injected with NS, PECE, SAHA, DDP and (Fig. 6A, p < 0.05). Therefore, SAHA-DDP, especially SAHA-DDP/PECE could led to reduced cell proliferation and increased apoptosis in vivo, which associated with inhibition of tumor growth.

To further determine the apoptotic mechanism in the protein level in vivo, western blot assay was performed. The result showed that the protein level of p53, cleaved Caspase-8, cleaved caspase-9 and cleaved caspase-3 in the tumor tissue of the SAHA-DDP/PECE treated mice much higher than the other groups (Fig. 6B). These data indicated that the observed cytotoxic effects of SAHA-DDP/PECE on HSC-3 tumor xenografts in nude mice also seemed to be mediated by the efficient induction of apoptotic mechanism just like our results in vitro [32].

## Discussion

In this study, we provided a novel chemotherapy protocol for oral squamous cell carcinoma. The PECE thermosensitive hydrogel have showed promising application as a chemotherapeutics controlled drug delivery [24]. We combined SAHA with DDP and utilized the SAHA-DDP/PECE complexes to induce an antitumor activity against the oral squamous cell carcinoma xenografts in a therapeutic setting. We found that the PECE hydrogel could be a novel delivery system and PECE hydrogel delivering DDP and SAHA had promising application in cancer chemotherapy.

DDP has a powerful therapeutic effect against oral carcinoma, but the resistance of tumor cells to DDP and dose-related toxicity remain two of the most important problems in the chemotherapy of clinical OSCC [32]. When the DDP dose was too high, the side-toxic effect was enhanced; when the DDP dose was low, the anticancer effect was too low. Researchers have been seeking a combinative treatment regimen to improve the effect of chemotherapy, so combination therapy using two or more chemotherapeutic agents was one of the most common strategies used in current oncology. SAHA was a newly developed HDAC inhibitor which could alter gene transcription and expression profile involved in cell-cycle regulation, tumor suppression, differentiation, and apoptosis [33]. It did this by silencing some tumor suppressor genes, as well as other genes that are responsible for cell cycle progression, cell proliferation, programmed cell death, and differentiation. Thus, blocking histone deacetylation may allow the body to block this tumor growth and prevent progression.

We have reported that low concentrations of SAHA synergized with DDP in combination therapy to induce a level of cytotoxicity and apoptosis in both Tca8113 and KB cell lines greater than that mediated by either agent alone, or that predicted by an additive model [32]. Previously, some researchers also have reported that the combination of SAHA with DDP possessed synergistic cytotoxicity against the other human cancer cells [34]–[38]. SAHA mediated the cleavage and activation of the pro-apoptotic Bcl-2 family member Bid. Furthermore, diverse apoptosis-associated proteins, including p53, cytochrome C and caspase-3 were involved in the induction of apoptosis. Their results suggest that concurrent treatment with SAHA enhances tumor cell sensitivity to subtoxic doses of DDP. Though this may be regarded as a novel strategy for treatment of cancer, it has not been detected in vivo. Nevertheless, further studies including generating OSCC xenograft mouse model should be conducted to confirm the in vitro results.

To design a better combinative chemotherapeutic regimen in vivo, there should be a better focus on cell killing and lower systemic toxicity. The selection of vectors always played an important role in the succession of chemotherapy protocol. And strategies such as sustained and localized therapy may have the potential to improve the efficacy of medical treatment. A kind of biodegradable and injectable poly (ethylene glycol)-poly (“-caprolactone)-poly (ethylene glycol) (PEG-PCL-PEG, PECE) triblock copolymer, can be used as a delivery vector to load chemotherapeutic drugs for intratumoral infusion chemotherapy. PECE hydrogel we have prepared in this study would be an ideal controlled drug delivery system for SAHA and DDP. As our previously study, a hydrogel solution (30 wt%, 0.5 mL) was subcutaneously injected into KunMing mice. After day 1, day 3, day 7, and day 14 following subcutaneous injection, and the opaque gel maintained its integrity in the period of observation. The size of gel decreased during degradation, and at day 14, the gel almost disappeared.Therefore, PECE hydrogel we have prepared in this study could sustain more than 14 days in situ [39], [40]. It was a free-flowing sol at room temperature or below critical gelation temperature became gel at body temperature. This sol-gel transition behavior of PECE hydrogel gives the advantage of simply mixing it with pharmaceutical agents at low temperature in which it then becomes an in situ gel-forming controlled drug delivery system in vivo at body temperature about 37°C [41], [42]. The previous results strongly support that PECE hydrogel can endue the delayedrelease properties and increased permeability of drugs. For intratumoral chemotherapy, prolonged stay and increased permeability of drugs are helpful for better disease control. In fact, the SAHA-DDP/PECE hydrogel system did show significantly increased therapeutic effects for OSCC. SAHA-DDP/PECE hydrogel system with direct intratumoral injections may be a useful method in treatment for oral cancer and other solid tumors.

We then investigated the possible application of this drug delivery system for the treatment of experimental OSCC by administrating the DDP and SAHA loaded PECE hydrogel peritumorally of OSCC-bearing mice. Our experimental results demonstrated that such a strategy as this allows the carrier system to show a sustained release of DDP and SAHA in vivo as well as improved therapeutic effects compared with a simple additive therapeutic effect of SAHA-DDP on murine OSCC. Furthermore, peritumorally administration of the SAHA-DDP/PECE was well tolerated and showed with no observable toxicity.

In conclusion, our data showed that intratumoral delivery of SAHA-DDP/PECE system was an efficient way to transfer chemical drugs to the tumor. And the administration of SAHA-DDP/PECE complexes led to the significant inhibition tumor grows of OSCC with no conspicuous systemic toxicity. So the treating oral squamous cell carcinoma by the PECE hydrogel delivered DDP and SAHA may be a new and interesting cancer chemotherapy protocol. SAHA-DDP/PECE hydrogel system with direct intratumoral injections may be a useful method in treatment for oral cancer and other solid tumors.
